# Impact of previous transurethral prostate surgery on health-related quality of life after radical prostatectomy: Does the interval between surgeries matter?

**DOI:** 10.1007/s00345-020-03327-4

**Published:** 2020-06-29

**Authors:** Michael Chaloupka, Franka Figura, Philipp Weinhold, Friedrich Jokisch, Thilo Westhofen, Paulo Pfitzinger, Robert Bischoff, Giuseppe Magistro, Frank Strittmatter, Armin Becker, Steffen Ormanns, Boris Schlenker, Alexander Buchner, Christian G. Stief, Alexander Kretschmer

**Affiliations:** 1 Department of Urology, Ludwig-Maximilians-University, Marchioninistrasse 15, 81377 Munich, Germany; 2 Department of Pathology, Ludwig-Maximilians-University, Munich, Germany

**Keywords:** EORTC QLQ-C30, Radical prostatectomy, Robot-assisted radical prostatectomy, Health-related quality of life, Transurethral resection of the prostate, Laser enucleation of the prostate, Laser vaporization of the prostate

## Abstract

**Purpose:**

To assess the impact of previous transurethral surgery for benign prostate enlargement (BPE) and time interval between procedures on functional outcomes and health-related quality of life (HRQOL) after radical prostatectomy (RP).

**Methods:**

A propensity score-matched patient cohort [*n* = 685, (513 without previous BPE surgery, 172 with BPE surgery)] was created and HRQOL was pre- and postoperatively assessed using validated questionnaires (EORTC QLQ-C30). Urinary continence was measured via ICIQ-SF questionnaire and pad usage. Multivariable analysis included binary logistic and Cox regression models (*p* < 0.05).

**Results:**

Median follow-up was 18 months. There was no significant difference in recurrence-free survival in multivariate analysis (HR 0.66, 95%CI 0.40–1.07, *p* = 0.093). We observe higher mean ICIQ-SF scores (5.7 vs. 8.2, *p* < 0.001) and daily pad usage (1.3 vs. 2.5, *p* < 0.001), and decreased continence recovery (OR 0.46, 95%CI 0.30–0.71, *p* < 0.001) for patients with BPE surgery. Postoperative general HRQOL scores were significantly lower for patients with previous BPE surgery (70.6 vs. 63.4, *p* = 0.003). In multivariate analysis, continence recovery (OR 5.19, 95%CI 3.10–8.68, *p* < 0.001) but not previous BPE surgery (0.94, 0.57–1.54, *p* = 0.806) could be identified as independent predictors of good general HRQOL. There was no significant correlation between time interval between both surgeries and continence (*p* = 0.408), and HRQOL (*p* = 0.386) outcomes.

**Conclusions:**

We observe favourable continence outcomes for patients without previous BPE surgery. Our results indicate that RP can be safely performed after transurethral BPE surgery, regardless of the time interval between both interventions.

## Introduction

Transurethral resection of the prostate (TUR-P) is the gold standard procedure in the management of refractory bladder outlet obstruction due to benign prostate enlargement, even though laser ablative transurethral procedures have become increasingly popular in recent years [1]. Radical prostatectomy (RP), on the other hand, represents an important cornerstone of the treatment of localized as well as locally advanced prostate cancer (PC) [2].

To date, there are data from several studies that investigated the effect of previous TUR-P on oncological as well as functional outcomes after RP, providing conflicting results [3–8]. In addition, generalizability of the current evidence is hampered by the fact that some studies analyzed historic cohorts [3, 4] or focused on a single surgical technique only [3, 4, 7]. Importantly, none of these studies evaluated health-related quality of life (HRQOL) and did not give information regarding the effect of the time interval between transurethral surgery and radical prostatectomy. To address these potential shortcomings, we created a large propensity score-matched cohort of contemporary patients that underwent RP in one experienced tertiary care centre and evaluated the impact of any type of previous transurethral BPE surgery as well as the time interval between both surgeries on patient-reported outcomes with a focus on HRQOL.

## Patients and methods

### Patient population, study design, and data assessment

To be eligible for the current study, patients had to fulfil the following inclusion criteria: ≤ pT3, no clinical lymph-node involvement, no clinical indication for metastatic disease based on preoperative bone scan or CT scan, and surgery performed by experienced surgeons with a minimum of 50 previous cases.

Between September 2013 and September 2019, 3436 radical prostatectomies [*n* = 2205 open retropubic RP, *n* = 1231 robot-assisted laparoscopic RP] have been performed in one tertiary care centre. After approval by an institutional review board, patient-reported outcomes were prospectively retrieved preoperatively as well as postoperatively. Hereby, questionnaires were sent per mail to eligible patients. Erectile dysfunction was assessed via the validated International Index of Erectile Function (IIEF5) questionnaire. Good erectile function was defined as IIEF-5 score of ≥ 18.

Patients that met all inclusion criteria were retrospectively selected and, consequently, a propensity score matching including the variables “age at prostatectomy” as well as “prostate volume based on histopathological specimen”, and “pT stage” was performed in a 1:3 fashion. Hereby, a matched cohort of 685 patients (*n* = 172 with previous BPE surgery, *n* = 513 without previous BPE surgery) was created and further analyzed.

Urinary continence was assessed using the International Consultation on Incontinence Questionnaire in its short form (ICIQ-SF). The ICIQ-SF is a three-item validated questionnaire. The total score ranges from 0 to 21, with higher scores indicating greater severity of urinary incontinence [9].

HRQOL was assessed using the EORTC QLQ-C30 questionnaire. The primary endpoint “general HRQOL” was assessed based on the global health status (GHS) domain of the QLQ-C30 questionnaire (questions 29 and 30) following current EORTC instructions [10]. Following Snyder et al. [11], good general HRQOL was defined as GHS of ≥ 70. For GHS, higher scores represent better general HRQOL. For QLQ-C30 functioning scores, higher scores represent a better functioning. For QLQ-C30 symptoms scores, higher scores represent greater impact of the respective symptom.

### Statistical analysis

As indicated above, a propensity score matching was performed and a matched patient cohort was created. Comparisons of patient-reported outcomes as well as EORTC QLQ-C30 subdomains between both subgroups were performed using Kruskal–Wallis analysis of variance and post hoc testing whenever denoted. For categorical data, Fisher’s exact test and Chi-square test were used. Primary endpoint for univariate and multivariable analyses was good general HRQOL at the respective time point based on a GHS score of ≥ 70, following previously published cut-off values [11]. For multivariable analysis, binary logistic as well as Cox regression models were used. Here, the number of events was defined as the number of patients with a GHS score of 70 or more. For univariate survival analyses, Kaplan–Meier curves were generated and log-rank testing was performed. For correlation of continuous parameters, Spearman’s rank correlation was used. All statistical analyses were performed using SPSS V26.0 (IBM, Armonk, NY, USA). A *p* value of < 0.05 was considered to be statistically significant.

## Results

### Perioperative patient characteristics and oncological outcomes

Detailed patient characteristics of the unmatched patient cohort are summarized in Table 1. Briefly, mean prostate volume was significantly higher for the patient subgroup without previous BPE surgery (57.6 vs. 49.4 ml, *p* = 0.001).

**Table 1:** Pre- and postoperative characteristics of patients included in the current study

	Unmatched cohort			Matched cohort		
	No BPE surgery	BPE surgery	*p*	No BPE surgery	BPE surgery	*p*
No. of patients	3259	177		513	172	
Age (years; mean ± SD)^a^	65.8 ± 8.1	66.5 ± 7.9	0.668	65.8 ± 8.0	66.4 ± 8.0	0.375
BMI (kg/m^2^; mean ± SD)	27.0 ± 6.8	26.7 ± 3.1	0.715	26.7 ± 3.3	26.4 ± 3.3	0.357
PSA preop. (ng/ml; mean ± SD)	17.2 ± 41.1	15.8 ± 23.2	0.933	15.1 ± 40.1	12.9 ± 17.5	0.048
Prostate volume (ml; mean ± SD)^a^	57.6 ± 24.2	49.4 ± 14.2	0.001	52.0 ± 21.6	50.2 ± 20.6	0.794
Gleason score [*n* (%)]						
6	350 (10.7)	31 (17.5)	0.002	70 (13.6)	30 (17.4)	0.093
7a	1191 (36.5)	42 (23.7)		191 (37.2)	46 (26.7)	
7b	728 (22.3)	40 (22.6)		109 (21.2)	39 (22.7)	
8	394 (12.1)	19 (10.7)		65 (12.7)	19 (11.0)	
9	520 (16.0)	33 (18.6)		72 (14.0)	32 (18.6)	
10	47 (1.4)	6 (3.4)		6 (1.2)	6 (3.5)	
pT stage [*n* (%)]^a^						
pT2	1868 (57.3)	98 (55.4)	0.512	280 (54.6)	94 (54.7)	1.000
pT3a	710 (21.8)	34 (19.2)		126 24.6)	41 (23.8)	
pT3b	660 (20.3)	44 (24.9)		107 (20.9)	37 (21.5)	
pT4	21 (0.6)	1 (0.6)		–	–	
Lymph-node involvement [*n* (%)]	378 (11.6)	21 (11.8)	0.866	55 (10.7)	19 (11.0)	0.912

*BPE *benign prostate enlargement, *BMI *body mass index, *PSA *prostate-specific antigen, *SD *standard deviation

^a^Propensity score-matched variables

In a next step, a matched cohort was created as described above. Detailed patient characteristics can be found in Table 1. We did not find statistically significant differences regarding tumour stage (*p* = 1.000), age (*p* = 0.375), and prostate volume (*p* = 0.794). Of note, mean PSA levels were significantly higher in the subcohort of patients without previous BPE surgery (15.1 vs. 12.9, *p* = 0.048).

Median follow-up was 18 months (3–351) for the matched cohort. Follow-up was available for 127 (73.8%) patients with previous BPE surgery and 379 (73.9%) patients without previous BPE surgery. Regarding the diagnosis of PC, 120 (69.8%) patients with previous BPE surgery were diagnosed by prostate biopsy, the remaining ones were scheduled for RP due to incidental PC diagnosis during the desobstruction procedure.

**Fig. 1:**
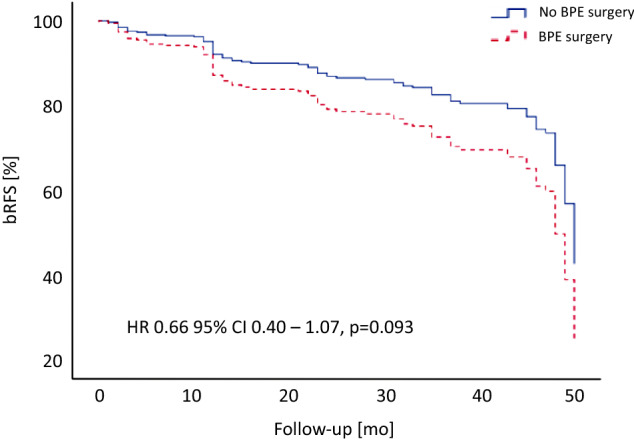
Biochemical recurrence-free survival (bRFS) for patients with (red) and without (blue) previous transurethral surgery for benign prostate enlargement (BPE)

The operation time (*p* = 0.976) as well as intraoperative blood loss (*p* = 0.080) were not significantly different between both subgroups. In addition, positive surgical margin rate for pT2 and pT3 tumours did not differ significantly [31.6% (no BPE surgery) vs. 34.5% (BPE surgery)]. In multivariate Cox regression analysis stratified for Gleason score and positive surgical margin status, no statistically significant differences were observed for biochemical recurrence-free survival (HR 0.66 95% CI 0.40–1.07, *p* = 0.093; Fig. 1).

### Functional outcomes

Preoperative as well as postoperative functional outcomes are summarized in Table 2. Briefly, we observed significantly higher preoperative rates of IIEF-5 scores  ≥ 18 in 40.8% (no BPE surgery) vs. 29.1% (BPE surgery) of the patients (*p* = 0.018). Postoperatively, 12.9% vs. 6.3% of the respective patient subgroups had IIEF-5 scores of ≥ 18 (*p* = 0.051). Regarding urinary continence, we found significantly higher mean ICIQ-SF scores (5.7 vs. 8.2, *p* < 0.001) and higher mean daily pad usage (1.3 vs. 2.5, *p* < 0.001) for patients with previous BPE surgery. Continence recovery, defined as use of up to one security pad per 24 h, was reached by 71.2% in patients without previous BPE surgery, and 52.1% of patients with previous BPE surgery (*p* < 0.001). In multivariate analysis stratified for age and prostate volume, previous BPE surgery could be confirmed as an independent risk factor for decreased continence recovery (OR 0.46, 95% CI 0.30–0.71, *p* < 0.001).

**Table 2:** Functional outcomes after a median follow-up of 12 months

	T0			Follow-up		
	No BPE surgery	BPE surgery	*p* value	No BPE surgery	BPE surgery	*p* value
Erectile function						
IIEF-5 score (mean ± SD)	11.2 ± 10.1	8.3 ± 9.8	< 0.001	5.7 ± 7.7	3.1 ± 5.7	< 0.001
IIEF-5 score ≥ 18 (%)	40.8	29.1	0.018	12.9	6.3	0.051
Urinary continence						
ICIQ-SF score (mean ± SD)	0.9 ± 2.5	2.5 ± 4.3	< 0.001	5.7 ± 5.2	8.2 ± 6.3	< 0.001
Daily pad usage (mean ± SD)	n.a	n.a	n.a	1.3 ± 1.9	2.5 ± 3.2	< 0.001
Continence recovery (%)	n.a	n.a	n.a	71.2	52.1	0.001

*BPE *benign prostate enlargement, *ICIQ-SF *International consultation of incontinence questionnaire short form, *IIEF-5 *international index of erectile function, *SD *standard deviation

### Health-related quality of life

**Fig. 2:**
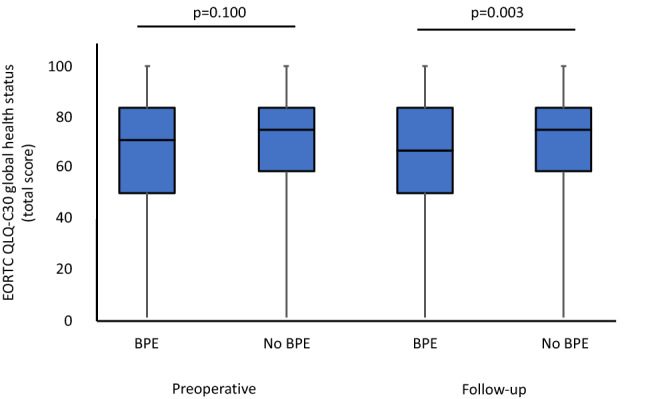
General health-related quality of life based on the QLQ-C30 global health status pre- (T0) and postoperatively (*BPE *benign prostate enlargement)

Health-related quality of life was assessed using the validated EORTC QLQ-C30 questionnaire. Analysis of functioning and symptoms subdomains as well as financial difficulties and general HRQOL based on GHS is summarized in Table 3. Preoperatively, no statistically significant differences in financial difficulty and functioning scales were observed. Regarding preoperative symptoms scale, there were significantly higher constipation scores in the BPE surgery subgroup (5.8 vs. 11.8, *p* < 0.001). Based on GHS, 50.0% (previous BPE surgery) and 56.8% (no previous BPE surgery, *p* = 0.197) could be classified as “good general HRQOL”. We did not observe statistically significant differences in mean GHS scores between patients with and without previous BPE surgery (71.6 vs. 68.0, *p* = 0.100; Fig. 2, Table 3).

**Table 3:** Preoperative (T0) and postoperative health-related quality of life outcomes based on the validated QLQ-C30 questionnaire

	Mean (SD) EORTC QLQ C30 score					
	T0			Follow-up		
	No BPE surgery	BPE surgery	*p*	No BPE surgery	BPE surgery	*p*
Symptome scale						
Dyspnoea	9.3 (20.2)	6.6 (19.2)	0.061	14.1 (25.8)	10.5 (21.1)	0.318
Pain	10.5 (21.7)	13.5 (24.5)	0.192	14.3 (26.3)	14.4 (25.2)	0.671
Fatigue	16.8 (24.5)	16.0 (21.0)	0.846	24.0 (25.7)	26.0 (23.3)	0.216
Insomnia	12.5 (23.8)	15.2 (24.0)	0.135	17.7 (28.3)	26.1 (31.9)	0.011
Appetite loss	4.2 (13.1)	6.3 (18.6)	0.413	3.8 (13.0)	8.4 (21.3)	0.021
Nausea/vomiting	1.1 (5.5)	1.1 (5.7)	0.803	2.3 (8.0)	1.8 (5.7)	0.893
Constipation	5.8 (17.8)	11.8 (22.9)	< 0.001	12.0 (23.4)	13.7 (25.5)	0.593
Diarrhoea	6.3 (15.8)	6.9 (17.7)	0.969	10.4 (19.9)	12.8 (22.5)	0.302
Financial difficulty scale	4.5 (15.9)	6.1 (20.2)	0.712	7.3 (17.7)	8.5 (18.3)	0.554
Functioning scale						
Physical	93.8 (12.0)	92.6 (13.6)	0.274	89.5 (17.0)	85.3 (19.1)	0.024
Role	89.9 (21.8)	89.5 (20.6)	0.543	80.4 (25.3)	75.0 (28.4)	0.108
Cognitive	88.8 (17.9)	86.8 (20.8)	0.629	82.6 (22.3)	83.9 (20.1)	0.716
Emotional	73.8 (23.4)	74.5 (23.9)	0.696	77.1 (24.4)	70.5 (25.5)	0.013
Social	84.8 (23.3)	84.5 (21.8)	0.616	75.9 (26.7)	69.4 (30.6)	0.075
Global health status	71.6 (20.9)	68.0 (22.6)	0.100	70.6 (21.8)	63.4 (24.1)	0.003

*BPE *benign prostate enlargement, *SD *standard deviation

Postoperatively, analysis of the QLQ-C30 symptoms scale revealed significantly increased insomnia (17.7 vs. 26.1, *p* = 0.011) and appetite loss scores (3.8 vs. 8.4, *p* = 0.021) for patients with previous BPE surgery compared to the subgroup without previous BPE surgery. In addition, emotional functioning scores (77.1 vs. 70.5, *p* = 0.013) and physical functioning scores (89.5 vs. 85.3, *p* = 0.024) were significantly lower for patients with previous BPE surgery. Postoperatively, mean GHS scores were significantly lower for patients with previous BPE surgery (70.6 vs. 63.4, *p* = 0.003; Fig. 2, Table 3) with 40.8% (previous BPE surgery) and 52.0% (no previous BPE, *p* = 0.038) being classified as “good general HRQOL” based on previously published GHS cut-off values [11]. Analysis of net changes of HRQOL subscales compared to baseline values is summarized in Table 4. Briefly, we observed no significant changes in GHS for the subgroup without previous BPE surgery (− 1.0, *p* = 0.289). In contrast, a significant decrease for patients with previous BPE surgery (− 4.6, *p* = 0.004) was found.

**Table 4:** Net differences between preoperative and postoperative health-related quality of life outcomes based on the validated QLQ-C30 questionnaire

	Change in mean EORTC QLQ C30 scores from baseline			
	No BPE surgery	*p*	BPE surgery	*p*
Symptome scale				
Dyspnoea	4.8	0.001	3.9	0.008
Pain	3.8	0.004	0.9	0.970
Fatigue	7.2	< 0.001	10.0	< 0.001
Insomnia	5.2	0.024	10.9	< 0.001
Appetite loss	− 0.4	0.502	2.1	0.014
Nausea/vomiting	1.2	0.028	0.7	0.463
Constipation	6.2	< 0.001	1.9	0.088
Diarrhoea	4.1	0.007	5.9	0.007
Financial difficulty scale	2.8	0.030	2.4	0.257
Functioning scale				
Physical	− 4.3	0.001	− 7.3	< 0.001
Role	− 9.5	< 0.001	− 14.5	< 0.001
Cognitive	− 6.2	< 0.001	− 2.9	0.221
Emotional	3.3	0.014	− 4.0	0.019
Social	− 8.9	< 0.001	− 15.1	< 0.001
Global health status	− 1.0	0.289	− 4.6	0.004

*BPE *benign prostate enlargement

To address potential learning curve effects, a separate analysis was conducted for the two most experienced surgeons with > 1000 previous RP vs. the remaining surgeons. Hereby, we did not find statistically significant differences regarding continence recovery (74.4 vs. 69.8%, *p* = 0.410), IIEF-5 scores of ≥ 18 (17.5 vs. 12.2%, *p* = 0.321), and good general HRQOL based on QLQ-C30 GHS (46.8 vs. 49.4%, *p* = 0.664). In addition, we did not find significant differences in continence recovery (*p* = 1.000), erectile function recovery (*p* = 1.000), and good general HRQOL rates (*p* = 0.589) for patients who underwent previous HoLEP instead of TUR-P.

In multivariable analysis regarding the primary endpoint “good general HRQOL (defined as GHS score of 70 or more), we stratified BPE surgery subgroups by functional outcomes based on continence recovery and achievement of IIEF-5 scores of ≥ 18 (Table 5). Hereby, continence recovery (OR 5.19, 95% CI 3.10–8.68, *p* < 0.001) but not previous BPE surgery (0.94, 0.57–1.54, *p* = 0.806) could be identified as independent predictors of better postoperative general HRQOL.

**Table 5:** Multivariable analysis regarding the primary endpoint, good general health-related quality of life (HRQOL)”, defined as QLQ-C30 global health status score of at least 70

Predictive feature for good HRQOL	Regression coefficient	Odds ratio	95% CI	*p* value
Previous BPE surgery (yes vs. no)	− 0.62	0.94	0.57–1.54	0.806
IIEF-5 18 or more (yes vs. no)	0.65	1.91	0.86–4.24	0.110
Continence recovery (yes vs. no)	1.65	5.19	3.10–8.68	< 0.001

*BPE *benign prostate enlargement, *CI *confidence interval, *IIEF-5 *International index of erectile function questionnaire

### Time interval between transurethral BPE surgery and radical prostatectomy

Information regarding the time interval between BPE surgery and RP was available for 144 of 172 patients (83.7%). Median time interval between the BPE surgery and RP was 27 months (1–443). Time interval was < 12 months for 56 patients (38.9%) and ≥ 12 months for 88 patients (61.1%).

**Fig. 3:**
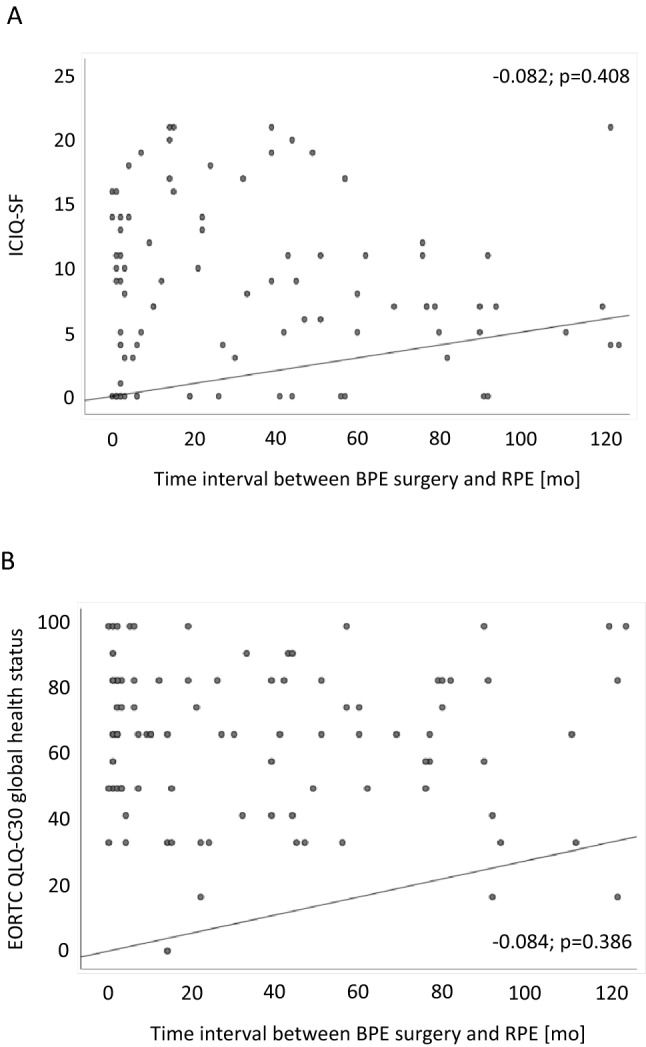
Spearman’s rank correlation regarding the impact of the time interval between transurethral benign prostate enlargement (BPE) surgery and radical prostatectomy (RPE) on urinary continence outcomes (**a**) and general health-related quality of life (**b**) (*ICIQ-SF *International consultation of incontinence questionnaire short form)

Using Spearman’s rank correlation, we did not observe a significant correlation between time interval of transurethral BPE surgery and RP for urinary continence based on validated ICIQ-SF scores (correlation coefficient − 0.082, *p* = 0.408; Fig. 3a) as well as for QLQ-C30 GHS scores (correlation coefficient − 0.084, *p* = 0.386; Fig. 3b). In line, we did not observe statistically significant differences in continence recovery rates between patients with less compared to more than 12 months of time interval between both surgeries (66.7 vs. 48.4%, *p* = 0.131).

## Discussion

In the current analysis, we provide data from a large contemporary propensity score-matched patient cohort and found favourable functional outcomes for patients that did not undergo previous BPE surgery and confirmed previous BPE surgery as an independent risk factor for decreased continence recovery in multivariable analysis. These findings are in line with results of recently published meta-analyses that found worse functional outcomes for patients with previous TUR-P [12, 13]. In the largest patient cohort to date, the authors found a significantly increased risk for urinary incontinence 3 months as well as 12 months after RP as well as worse erectile function recovery rates [8]. However, the continence rates that are reported in the current study have to be interpreted with caution. First, it has to be emphasized that the patient cohort analysed in the current study is a relatively high-risk patient population and a majority of patients were categorized in high-risk ISUP grade groups. This has to be kept in mind when functional outcomes of the current study are compared with previously reported continence rates. In addition, definitions of continence as well as methods of continence function data retrieval vary between the currently available studies. In the current study, we provide continence data based on pad usage as well as the validated ICIQ-SF questionnaire to provide robust and reproducible continence data. Appropriate assessment of functional outcomes is important for preoperative patient counseling even if there are no adequate alternative therapies available, since it has been shown that well-perceived patient education has the potential to improve postoperative patient satisfaction following RP [14].

It has been postulated that increased inflammation and tissue fibrosis lead to more challenging surgical procedures, resulting not only in decreased functional but also oncological outcomes. Thus, it could be anticipated that a shorter period between BPE surgery and RP might lead to more difficult surgical procedures and ultimately leads towards decreased functional as well as oncological outcomes. For instance, Jaffe et al. analyzed the outcomes of 118 patients that underwent laparoscopic RP following TUR-P and found higher positive surgical margin rates for patients with previous TUR-P [3]. This is in line with the results of a recent meta-analysis by Li et al. [13]. However, there are also studies who failed to observe differences in positive surgical margin and biochemical recurrence rates [8]. Analogously, we did not observe any significant differences in positive surgical margin rates and biochemical recurrence-free survival for patients with previous BPE surgery in univariate and multivariate analysis.

In the absence of profound oncological data and conflicting results of functional outcomes, soft endpoints such as HRQOL are essential to assess potentially harmful effects of previous transurethral BPE surgeries. In addition to the results of previous studies, our study is the first to provide pre- as well as postoperative HRQOL data. For other uro-oncological entities, it has already been shown that preoperative HRQOL is an essential contributor in guidance of therapy decision-making [15] and it can be anticipated that this is also true for pre-RP patient counselling. While we observed significantly higher rates of good general HRQOL in univariate analysis, we were not able to confirm previous BPE as an independent risk factor for decreased general HRQOL in multivariate analysis stratified for continence recovery and IIEF-5 scores. Future studies with larger cohorts are, therefore, needed to confirm these preliminary results. In addition to general HRQOL scores, we provide detailed data from HRQOL subscales such as functioning and symptoms scores.

Another important novelty of the current study is the implementation of the time axis in the analysis of RP outcomes following transurethral BPE surgery. Interestingly, we did not observe any significant correlation between the time interval between BPE surgery and RP on continence as well as general HRQOL outcomes, and ICIQ-SF scores. While it can be hypothesized that inflammation is reduced over a longer time course, fibrosis-related effects might even increase within a longer time interval between both surgeries. Our results indicate that RP can be safely performed after transurethral BPE surgery, regardless of the time interval between both interventions.

The current study is not devoid of limitations. In addition to the limitations that are inherent to retrospective analyses in general, we use the non-prostate-specific EORTC QLQ-C30 questionnaire to address patients HRQOL. However, despite being not prostate cancer specific, this questionnaire provides robust results that can be compared with other entities as well as surgical procedures. To compensate the lack of domains that specifically address urinary and sexual symptoms, the validated IIEF-5 and ICIQ-SF questionnaires have been implemented in our analysis. Assessing HRQOL through validated questionnaires is advantageous in terms of generalizability and reproducibility of results but implies the important question whether statistically significant differences translate into clinically relevant differences. Even though this question cannot be adequately answered to date, a potential benefit of the QLQ-C30 questionnaire is that previously published and frequently used clinically relevant cut-off values are available to rely on [11]. Furthermore, the relatively short median follow-up of 18 months has to be addressed as another potential limitation of the current study and further studies with longer follow-up are needed in order to adequately address the impact of previous surgical desobstruction on biochemical recurrence-free survival after RP. In addition, it has to be emphasized that the subcohort with previous BPE surgery is still relatively small compared to their non-BPE surgery counterpart, which ultimately may lead to underpower and an increased probability of type II errors.

Finally, even though the current study is the first to report data from non-TUR-P surgically desobstructed patients, these subgroups are still small and larger, adequately powered studies are needed to further evaluate outcomes in this respective patient cohort.

## Conclusions

In summary, we provide data from a large and well-balanced contemporary propensity score-matched patient cohort and focus on the impact of previous BPE surgeries on HRQOL outcomes following RP with an emphasis on the effect of the time interval between both procedures. After a median follow-up of 18 months, we found significantly decreased continence rates after previous BPE surgery as well as significantly higher postoperative general HRQOL scores for patients without BPE surgery in univariate analysis without statistically significant differences in multivariate analysis. We did not observe any significant impact of the time interval between both procedures indicating that RP can be safely performed regardless of the respective time interval.

## Abbreviations

BPE: Benign prostate enlargement
GHS: Global health status
HRQOL: Health-related quality of life
ICIQ-SF: International consultation on incontinence (short form)
ISUP: International Society of Urological Pathologists
IIEF-5: International Index on Erectile Function
ORP: Open radical prostatectomy
RARP: Robot-assisted radical prostatectomy
RP: Radical prostatectomy
TUR-P: Transurethral resection of the prostate

## References

[1] Gravas S, Cornu JN, Gacci M, Gratzke C, Herrmann TRW, Mamoulakis C, et al. (2020) EAU Guidelines on Management of Non-Neurogenic Male Lower Urinary Tract Symptoms (LUTS), incl. Benign Prostatic Obstruction (BPO) 2020. European Association of Urology Guidelines 2020 Edition. Presented at the EAU Annual Congress Amsterdam 2020. European Association of Urology Guidelines Office, Arnhem

[2] Mottet N, van den Bergh RCN, Briers E, Cornford P, De Santis M, Fanti S, et al. (2020) EAU-ESTRO-ESUR-SIOG Guidelines on Prostate Cancer 2020. European Association of Urology Guidelines 2020 Edition. Presented at the EAU Annual Congress Amsterdam 2020. European Association of Urology Guidelines Office, Arnhem

[3] Jaffe J, Stakhovsky O, Cathelineau X, Barret E, Vallancien G, Rozet F (2007). Surgical outcomes for men undergoing laparoscopic radical prostatectomy after transurethral resection of the prostate. J Urol.

[4] Colombo R, Naspro R, Salonia A, Montorsi F, Raber M, Suardi N (2006). Radical prostatectomy after previous prostate surgery: clinical and functional outcomes. J Urol.

[5] Martin AD, Desai PJ, Nunez RN, Martin GL, Andrews PE, Ferrigni RG (2009). Does a history of previous surgery or radiation to the prostate affect outcomes of robot-assisted radical prostatectomy?. BJU Int.

[6] Palisaar JR, Wenske S, Sommerer F, Hinkel A, Noldus J (2009). Open radical retropubic prostatectomy gives favourable surgical and functional outcomes after transurethral resection of the prostate. BJU Int.

[7] Hung CF, Yang CK, Ou YC (2014). Robotic assisted laparoscopic radical prostatectomy following transurethral resection of the prostate: perioperative, oncologic and functional outcomes. Prostate Int.

[8] Pompe RS, Leyh-Bannurah SR, Preisser F, Salomon G, Graefen M, Huland H (2018). Radical prostatectomy after previous TUR-P: oncological, surgical, and functional outcomes. Urol Oncol.

[9] Avery K, Donovan J, Peters TJ, Shaw C, Gotoh M, Abrams P (2004). ICIQ: a brief and robust measure for evaluating the symptoms and impact of urinary incontinence. Neurourol Urodyn.

[10] Aaronson NK, Ahmedzai S, Bergman B, Bullinger M, Cull A, Duez NJ (1993). The European Organization for Research and Treatment of Cancer QLQ-C30: a quality-of-life instrument for use in international clinical trials in oncology. J Natl Cancer Inst.

[11] Snyder CF, Blackford AL, Okuyama T, Akechi T, Yamashita H, Toyama T (2013). Using the EORTC-QLQ-C30 in clinical practice for patient management: identifying scores requiring a clinician's attention. Qual Life Res.

[12] Liao H, Duan X, Du Y, Mou X, Hu T, Cai T et al (2019) Radical prostatectomy after previous transurethral resection of the prostate: oncological, surgical and functional outcomes-a meta-analysis. World J Urol. 10.1007/s00345-019-02986-2 10.1007/s00345-019-02986-2 31679064

[13] Li H, Zhao C, Liu P, Hu J, Yi Z, Chen J (2019). Radical prostatectomy after previous transurethral resection of the prostate: a systematic review and meta-analysis. Transl Androl Urol.

[14] Kretschmer A, Buchner A, Grabbert M, Sommer A, Herlemann A, Stief CG (2017). Perioperative patient education improves long-term satisfaction rates of low-risk prostate cancer patients after radical prostatectomy. World J Urol.

[15] Singh V, Yadav R, Sinha RJ, Gupta DK (2014). Prospective comparison of quality-of-life outcomes between ileal conduit urinary diversion and orthotopic neobladder reconstruction after radical cystectomy: a statistical model. BJU Int.

