# Correction to: Impact of previous transurethral prostate surgery on health-related quality of life after radical prostatectomy: Does the interval between surgeries matter?

**DOI:** 10.1007/s00345-021-03754-x

**Published:** 2021-06-30

**Authors:** Michael Chaloupka, Franka Figura, Philipp Weinhold, Friedrich Jokisch, Thilo Westhofen, Paulo Pfitzinger, Robert Bischoff, Giuseppe Magistro, Frank Strittmatter, Armin Becker, Steffen Ormanns, Boris Schlenker, Alexander Buchner, Christian G. Stief, Alexander Kretschmer

**Affiliations:** 1 Department of Urology, Ludwig-Maximilians-University, Marchioninistrasse 15, 81377 Munich, Germany; 2 Department of Pathology, Ludwig-Maximilians-University, Munich, Germany

## Correction to: World Journal of Urology (2021) 39:1431–1438 10.1007/s00345-020-03327-4

The article “Impact of previous transurethral prostate surgery on health-related quality of life after radical prostatectomy: Does the interval between surgeries matter?” written by Chaloupka, M., Figura, F., Weinhold, P., Jokisch, F., Westhofen, T., Pfitzinger, P., Bischoff, R., Magistro, G., Strittmatter, F., Becker, A., Ormanns, S., Schlenker, B., Buchner, A., Stief, C.G. and Kretschmer, A. was originally published Online First without Open Access. After publication in volume 39, issue 5, pages 1431–1438 the author decided to opt for Open Choice and to make the article an Open Access publication. Therefore, the copyright of the article has been changed to © The Author(s) 2020 and the article is forthwith distributed under the terms of the Creative Commons Attribution 4.0 International License, which permits use, sharing, adaptation, distribution and reproduction in any medium or format, as long as you give appropriate credit to the original author(s) and the source, provide a link to the Creative Commons licence, and indicate if changes were made. The images or other third party material in this article are included in the article’s Creative Commons licence, unless indicated otherwise in a credit line to the material. If material is not included in the article’s Creative Commons licence and your intended use is not permitted by statutory regulation or exceeds the permitted use, you will need to obtain permission directly from the copyright holder. To view a copy of this licence, visit http://creativecommons.org/licenses/by/4.0.

The original article has been corrected.

